# iCCM data quality: an approach to assessing iCCM reporting systems and data quality in 5 African countries

**DOI:** 10.7189/jogh.09.010805

**Published:** 2019-06

**Authors:** Lwendo Moonzwe Davis, Kirsten Zalisk, Samantha Herrera, Debra Prosnitz, Helen Coelho, Jennifer Yourkavitch

**Affiliations:** 1 ICF, Rockville, Maryland, USA; 2 Save the Children, Washington, D.C., USA

## Abstract

**Background:**

Ensuring the quality of health service data is critical for data-driven decision-making. Data quality assessments (DQAs) are used to determine if data are of sufficient quality to support their intended use. However, guidance on how to conduct DQAs specifically for community-based interventions, such as integrated community case management (iCCM) programs, is limited. As part of the World Health Organization’s (WHO) Rapid Access Expansion (RAcE) Programme, ICF conducted DQAs in a unique effort to characterize the quality of community health worker-generated data and to use DQA findings to strengthen reporting systems and decision-making.

**Methods:**

We present our experience implementing assessments using standardized DQA tools in the six RAcE project sites in the Democratic Republic of Congo, Malawi, Mozambique, Niger, and Nigeria. We describe the process used to create the RAcE DQA tools, adapt the tools to country contexts, and develop the iCCM DQA Toolkit, which enables countries to carry out regular and rapid DQAs. We provide examples of how we used results to generate recommendations.

**Results:**

The DQA tools were customized for each RAcE project to assess the iCCM data reporting system, trace iCCM indicators through this system, and to ensure that DQAs were efficient and generated useful recommendations. This experience led to creation of an iCCM DQA Toolkit comprised of simplified versions of RAcE DQA tools and a guidance document. It includes system assessment questions that elicit actionable responses and a simplified data tracing tool focused on one treatment indicator for each iCCM focus illness: diarrhea, malaria, and pneumonia. The toolkit is intended for use at the national or sub-national level for periodic data quality checks.

**Conclusions:**

The iCCM DQA Toolkit was designed to be easily tailored to different data reporting system structures because iCCM data reporting tools and data flow vary substantially. The toolkit enables countries to identify points in the reporting system where data quality is compromised and areas of the reporting system that require strengthening, so that countries can make informed adjustments that improve data quality, strengthen reporting systems, and inform decision-making.

Quality of data refers to the degree to which the data collected measure what they were intended to measure. Data quality is a multi-dimensional concept, inclusive of several elements: accuracy, availability, completeness, confidentiality, integrity, precision, reliability, and timeliness [1,2]. Many factors can impact the quality of data collected, including inappropriate or inadequate data collection instruments and procedures, poor recording and reporting, and errors in data processing [3]. For routine health information data, data quality assessments (DQAs) play an important role in determining if data meet the quality required to support their intended use, identifying data quality challenges, and providing recommendations to improve the quality of data. DQAs should also assess data collection processes and data use [4]. DQAs are especially important as many monitoring systems fail to deliver data that are relevant, complete, timely, and accurate. Further, in the field of global health there has been a push for data-driven or evidence-based decision-making [5-11]. However, many program managers are ill-equipped and do not have the data needed for informed decision-making [5,12-15]. Even when data are available, their quality may be weak, limiting their usefulness for appropriate decision-making [13].

Community-based interventions are expanding in technical and geographic scope in an effort to improve health service coverage and equity. Evidence is needed to inform community-based programming, and to ensure its quality. As such, the quality of data generated at the community level is especially important. Integrated community case management (iCCM) is increasingly being used as a strategy to enable community health workers (CHWs) to diagnose and provide treatment for pneumonia, diarrhea, and malaria, the three major causes of childhood mortality [16,17]. Communities defined as hard-to-reach due to their limited access to a health facility typically employ an iCCM strategy. For iCCM interventions, routine data are vital to assess program performance and identify areas for improvement [13,14,18,19]. However, iCCM data face several data quality problems, both due to challenges specific to CHWs and because adding another level to the reporting system creates more opportunities for errors. CHWs, who generate iCCM data, have varied, but often limited, literacy and numeracy levels, limited time or tight timeframes to record and report data, limited resources, minimal training on data recording and reporting, poor physical infrastructure for submitting reports in a timely manner, and few incentives [14,20,21]. Errors in data recording and aggregation introduce further challenges in ensuring data quality [22]. DQAs play an important role in identifying and making recommendations to address some of these problems. However, literature and published guidance on how to conduct DQAs specifically for community-based interventions, such as iCCM programs, is limited [21,23]. Several studies have also noted the importance of documenting and describing the process used to conduct DQAs, especially for data originating from the community level [24].

The World Health Organization (WHO) launched the Rapid Access Expansion Programme (RAcE) in the Democratic Republic of Congo, Malawi, Mozambique, Niger and Nigeria in 2013. Under RAcE international non-governmental organizations (NGOs) and WHO supported Ministries of Health (MOH) to implement iCCM programs. The RAcE programme had two primary objectives: to catalyze the scale-up of community case management of malaria (CCMm) and iCCM, and to stimulate policy review and regulatory updates in each country on disease case management. Throughout the implementation of RAcE, ICF provided independent technical support for monitoring and evaluation activities, which included designing and conducting DQAs.

In this article, we describe the process used to create and adapt RAcE DQA tools and develop the iCCM DQA Toolkit, which enables countries to carry out regular and rapid DQAs. Drawing on our DQA protocols and reports, we also provide examples of how we used results to generate recommendations for project implementing partners and national-level stakeholders.

## METHODS

In 2013, ICF designed DQA tools to assess the quality of iCCM data and the iCCM data collection, reporting, and management system for RAcE. The DQA tools were adapted from the Global Fund’s DQA tool developed by MEASURE Evaluation for facility-based HIV and AIDS treatment programs to focus on iCCM data generated at the community level and iCCM data reporting and management system. Papers by Nyangara et al. 2018 and Yourkavitch et al. 2016 provide further details on the methods utilized for the RAcE DQAs [16,25]. In each of the six RAcE project areas, two DQAs were conducted one to two years apart (**Table 1**). The time between the first and second DQAs allowed grantees and project stakeholders to make modifications to the reporting system based on DQA findings and recommendations to improve data quality.

**Table 1:** Timing of RAcE DQAs

Project site	Round 1 DQA	Round 2 DQA
**Democratic Republic of Congo**	June 2014	September 2015
**Malawi**	January 2014	February 2016
**Mozambique**	June 2015	October 2016
**Niger**	April 2014	June 2015
**Nigeria (Abia State)**	October 2015	November 2016
**Nigeria (Niger State)**	October 2015	December 2016

RAcE – Rapid Access Expansion, DQA – data quality assessment

Although there were slight variations in the DQA methodology across project sites and sometimes between rounds, all of the DQAs had three primary objectives:

- To assess the effectiveness of the grantee data collection system, and identify any bottlenecks in the national health information system that affected grantee routine reporting
- To assess the integrity of project data, including CHW and supervisor registers, and data on services provided/case management, supervision and commodity stockouts
- To provide guidance and recommendations to grantees and national stakeholders in the generation of quality data to guide the implementation of the project and improve data quality

In each DQA, ICF evaluated data quality through several exercises including: mapping the data flow, tracing and verifying the data, and assessing the iCCM data reporting system in place. ICF also provided detailed recommendations based on the findings from each DQA. At the request for an external assessment by the WHO, ICF staff and in-country consultants conducted the DQAs, with logistic support and engagement of grantee staff. The DQAs utilized both quantitative and qualitative data collection methods to accomplish the aforementioned objectives. We briefed grantee, MOH, and other national stakeholder staff prior to DQA fieldwork and debriefed them with preliminary results immediately following fieldwork. Also at the request of WHO, ICF developed the iCCM DQA Toolkit, intended to be a resource for MOH staff. The toolkit includes data collection and analysis tools and accompanying guidance necessary to carry out routine DQAs.

## RESULTS

### Tool development process

**Figure 1:**
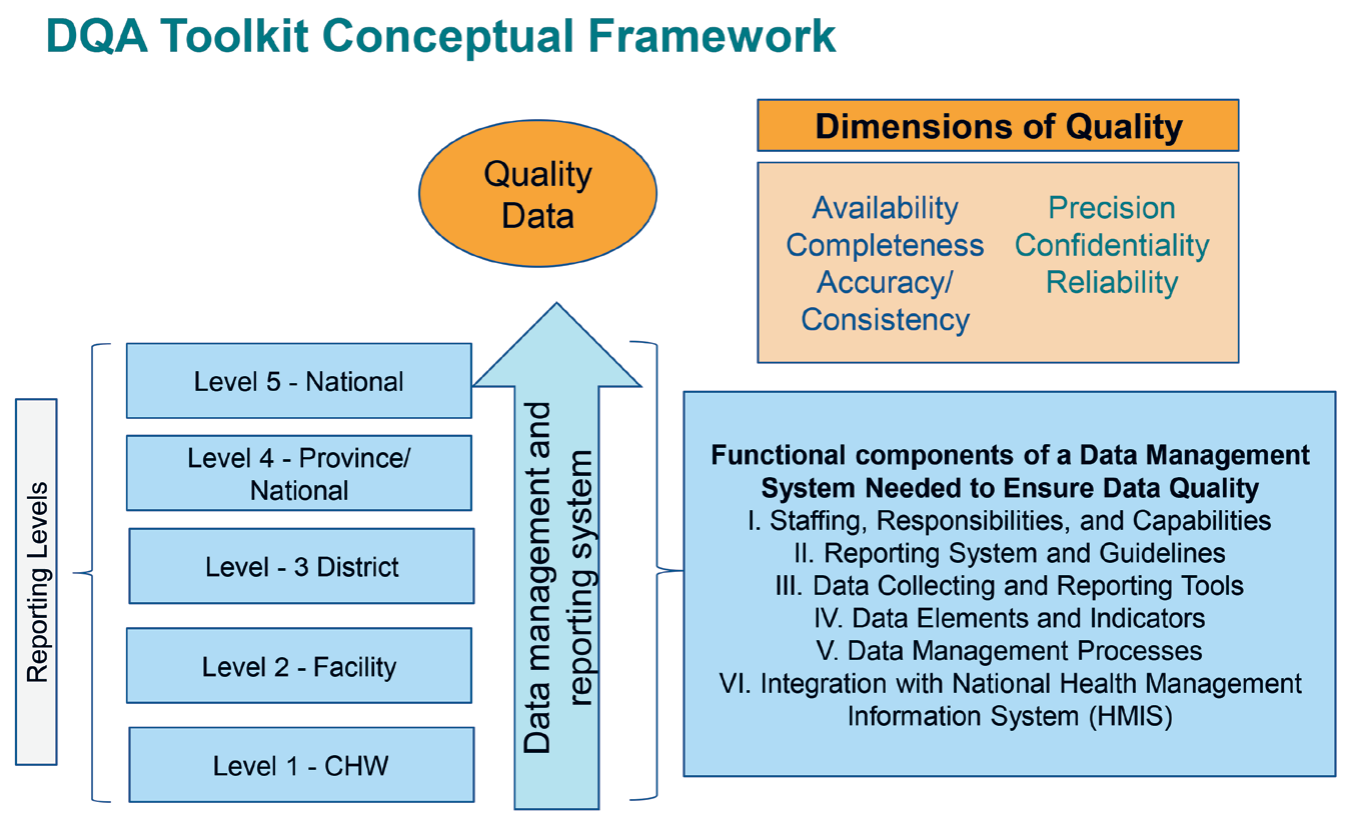
Rapid Access Expansion (RAcE) data quality assessment (DQA) toolkit conceptual framework adapted from MEASURE Evaluation, Data Quality Audit Tool, 2008.

ICF developed DQA tools to assess the iCCM data reporting system and to trace iCCM indicators through this system. We adapted the conceptualization and methodology for the RAcE DQAs from the Global Fund’s DQA for HIV and AIDS treatment programs [25] (**Figure 1**).

The design of the DQA tools was based on MEASURE Evaluation’s Excel-based tool to assess the quality of HIV/AIDS treatment data emanating from facility-based services [2]. However, to fit the purposes of the assessed iCCM programs, we modified the tool to focus on iCCM, extend data collection from the facility level to the community level, and incorporate qualitative data collection to complement findings from quantitative data. In modifying the MEASURE Evaluation tool, iCCM data flows were mapped using iCCM data collection and reporting tools and we reviewed grantees’ RAcE performance monitoring frameworks (PMFs) to determine which indicators or data fields to trace. After conducting the first DQA in Malawi, we developed customized paper “tracker forms” to record data extracted from iCCM registers and reporting forms, which we later entered into the Excel (Microsoft Inc, Seattle, WA, USA) tool.

### Mapping the data flow

**Figure 2:**
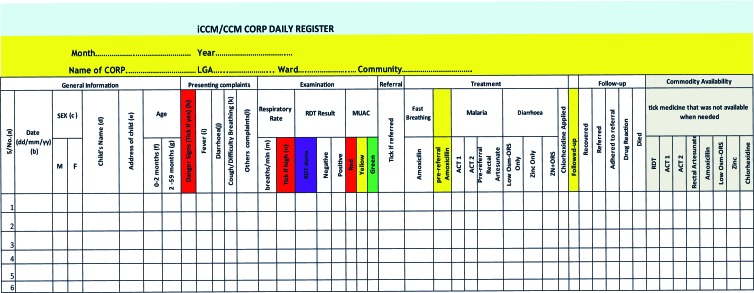
Sample community health worker (CHW) iCCM Registry, Nigeria.

Reporting systems varied by country and a critical step of adapting the tools was to understand the data flow. In DRC, Malawi, Mozambique and Niger, data flowed from the CHW or community level to the facility level, to an intermediate aggregation level (eg, district, province), and then in parallel to the central/national and grantee levels. However, in Nigeria there were no systems in place to report community-level data to the central or national level. Data from the community level in Nigeria were submitted to the grantees and available to the State Ministries of Health (SMOH). Because the DQAs were conducted to assess the RAcE grantees’ reporting systems, iCCM data were traced from the community level to the grantee level. Due to the variation of each data reporting system, it was critical to map out the systems to tailor the DQA tools. For instance, in the majority of RAcE project areas, CHWs generated monthly iCCM reports using the data in their iCCM registers and submitted them to the health facility level, but in two project areas, CHW supervisors generated comparable reports. In two RAcE project areas, CHW-generated data were aggregated at the facility level, but in the other four project areas, data were passed from the facility level to the next reporting level by CHWs. At this next reporting level, staff entered data into an electronic data management system, into a database, or into another paper form, depending on the RAcE project area, and sometimes depending on the location within the RAcE project area. **Figure 2** is an example of a CHW register from Nigeria (samples of other registers are available on the CCM Central Website: https://ccmcentral.com/resources-and-tools/tools-for-chws/).

### Indicators traced

As part of the DQA planning processes, we selected key iCCM indicators from the grantees’ PMFs to trace. The DQA tools were then adapted for each RAcE project area based on the indicators which were traceable through the data flow and the fields in the data collection and reporting tools. Selecting indicators, reviewing the data elements needed to calculate each indicator, and then ensuring that the iCCM data collection and reporting tools captured these data elements were critical steps in DQA planning. In some countries, this planning process revealed that reporting tools were not accurately capturing data elements needed to calculate iCCM indicators, and that consequently data grantees reported were inaccurate. Across all sites, we examined indicators related to treatment of diarrhea, malaria, and pneumonia over a three-month period that aligned with the grantees’ last completed RAcE reporting quarter. We also examined stockout and supervision indicators if the information was available in the reporting forms.

### Data collection methods

The DQA tools implemented a mixed-method approach consisting of qualitative and quantitative methods to assess data quality and the reporting system, summarized in **Table 2**.

**Table 2:** Summary of data collection tools, sources and outputs

	Qualitative methods	Quantitative methods
**Tool**	• Interview Guide* (Word Document)	• Data tracing and verification (Excel)
		• Systems assessment (Excel)
**Data Sources**	• Central level staff, intermediate aggregation level staff, CHW supervisor and CHW involved in iCCM data collection, management or reporting	• Data collection and reporting tools, including iCCM registers, paper reporting forms, and computerized data files
		• Central level staff, intermediate aggregation level staff and CHW supervisor involved in iCCM data collection, management or reporting
**Outputs**	• Interview notes	• Availability and completeness measures
		• Verification ratios*
		• Consistency ratios
		• Absolute counts and differences among data sources
		• Scorecard with average scores for each component across each level assessed

RAcE – Rapid Access Expansion, CHW – community health worker

*Included in RAcE DQAs but not included in the iCCM DQA Toolkit.

*Qualitative methods* were used to capture involvement of and perceptions from stakeholders across all levels of the reporting system and to assess how systems functioned. Specific areas of inquiry covered in the interview guide included data use, understanding and adequacy of data collection and reporting tools, training and supervisory support provided and received to complete data collection forms, and perceptions of workload. Qualitative data collection also enabled us to learn more about the data quality challenges associated with the collection, management, and reporting system(s) in place. We collected qualitative data through key informant interviews and document reviews. Although the specific types and numbers of individuals interviewed varied, the DQA team conducted interviews with individuals who were involved in iCCM data collection, reporting, or data management at each level of the reporting system. Across RAcE project areas, the DQA team interviewed one iCCM CHW supervisor and one randomly selected CHW at each sampled facility. Interviews were conducted using a semi-structured interview guide and were either audio recorded or documented with notes. Local consultants conducted interviews in the local languages or through a translator and ICF staff conducted other interviews in English, French, or Portuguese. The DQA team also conducted comprehensive reviews of all RAcE grantee and applicable iCCM data collection and reporting tools, and the data and information flow to assess their quality and to identify any potential bottlenecks or challenges.

*Quantitative methods* were used to assess the quality of the data collected at each reporting level with a focus on key project indicators. Data were collected and scored using the systems assessment tool, and CHW counts of treatments were verified and traced through the reporting system using the data tracing tool. The systems assessment included standardized questions across five dimensions as identified in **Figure 1**: (1) Monitoring and Evaluation (M&E) structures, functions, and capabilities, 2) indicator definitions and reporting guidelines, 3) data collection and reporting forms, 4) data management processes, and 5) links with the national reporting system. Although we standardized the questions across all RAcE project sites, depending on the systems, we excluded some questions that were not applicable. For instance, if RAcE project sites did not enter data into an electronic data management system or database at the sub-national reporting level, we excluded the questions about electronic data entry from the systems assessment for that level.

The DQAs used data tracing and verification to detect inconsistencies and unexpected values in the information reported. The DQAs also collected quantitative data to assess the availability and completeness of data collection and reporting forms. At each facility visited, the DQA team reviewed the iCCM registers for all CHWs who reported to that facility. The DQA team also reviewed any monthly reports that the CHWs submitted to the health facility. At the next level of the reporting system, the DQA team reviewed the reports from the facilities submitted to the sites visited at that level (eg, district, provincial or local government authority (LGA) health offices). At the grantee’s office, the DQA team reviewed the data that they received and the data in their project database, if they had one. Data for the traced indicators were extracted from the iCCM registers and reporting forms, then recorded on the paper tracker sheets and entered into the data tracing Excel tool.

### Sampling methodology

Understanding the reporting system and data flow was critical to the site selection for each DQA to ensure that sites at each level of the reporting system were included. In all RAcE project areas, CHWs reported to a facility-based supervisor. In order to select the sample, we needed a complete list of the program health facilities, including the number of supervisors at each facility and the number of CHWs who reported to each supervisor. We developed the sampling strategy implemented in the DQAs to be logistically feasible and to provide a snapshot of what was happening in each project area; it was not intended to be statistically representative, but rather to highlight key themes around data quality that needed to be addressed. The iCCM DQA Toolkit (available at http://ccmcentral.com/) discusses further sampling considerations, such as funding, timeframe for the assessment, and other logistical considerations.

Across the RAcE project areas, there were several considerations made for site selection. In some cases, we used multi-stage sampling to make the DQA fieldwork more feasible within a two to three week period. For example, in Mozambique’s second DQA, we randomly selected two of the four RAcE supported provinces; however, within those provinces we excluded districts that were unsafe and those that were not yet using the new reporting forms. Additionally in Mozambique, because of the geographic spread of RAcE project areas, we accounted for distance in the sampling to ensure feasibility of data collection. We then selected facilities based on having at least one active CHW for the entire reporting period. Back-up facilities were also selected in the event that one of the original facilities was inaccessible. In DRC, conflicts in one of the health zones forced suspension of implementation in that health zone and cut off access to other health zones that could only be reached by traveling through the conflict area. Therefore, we implemented a purposeful sampling approach. In all other RAcE project areas, we implemented a random selection approach.

A larger number of facilities was sampled in the first DQAs in Malawi and Niger. For logistic feasibility, we sampled eight facilities in subsequent DQAs, but the number of CHWs associated with each of the selected facilities varied (**Table 3**). Additionally, the number of CHWs whose data were verified and traced may have been less than the total number of CHWs who reported to the sampled facilities, if for example, on the day of the DQA team’s visit, a CHW did not report to or bring their iCCM registers to the facility. The DQA teams traced and verified for all CHWs present; however, they randomly selected one CHW at each facility for an interview. **Table 3** summarizes the number of facilities sampled for each DQA and the number and type of CHWs that reported to each sampled facility and were included in the DQA.

**Table 3:** Samples for first and second RAcE DQAs

RAcE Project and DQA	Number of nistricts, LGA, Health zones	Number of Facilities	Number of CHWs included in assessment	CHW cadre	
**DRC (DQA 1)**	4	8	60		*Relais Communitaire (RCom)*
**DRC (DQA 2)**	3	8	52		
**Malawi (DQA 1)**	4	10	52		Health Surveillance Assitant (HSA)
**Malawi (DQA 2)**	4	8	34		
**Mozambique (DQA 1)**	6	8	31		*Agente Polivalente Elementar* (APE)
**Mozambique (DQA 2)**	4	8	23		
**Niger (DQA 1)**	2	16	83*		*Relais Communitaire (RCom)*
**Niger (DQA 2)**	4	8	85		
**Nigeria – Abia State (DQA 1)**	4	8	67		Community-Oriented Resource Person (CORP)
**Nigeria – Abia State (DQA 2)**	4	8	48		
**Nigeria – Niger State (DQA 1)**	3	8	30		
**Nigeria – Niger State (DQA 2)**	6	8	61		

RAcE – Rapid Access Expansion, DQA – data quality assessment, LGA – local government authority, CHW – community health worker

*Only 83 of 146 RCom were included in the DQA. Others were ill, had dropped out of the CHW program, or were not available for other reasons.

### Systems assessment

**Figure 3:**
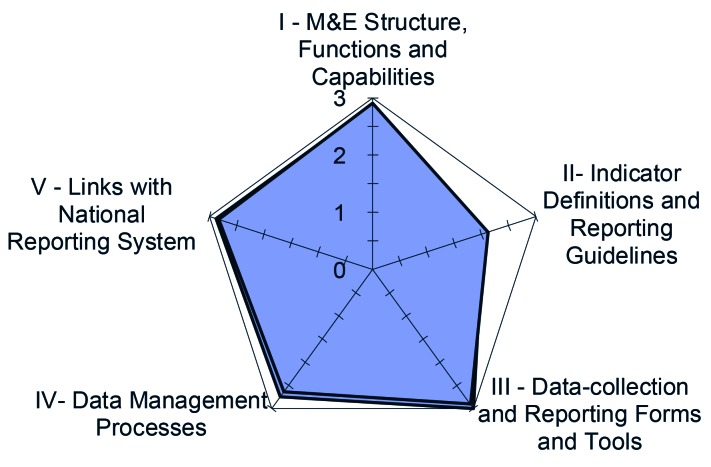
Example of overall Rapid Access Expansion (RAcE) data quality assessment (DQA) systems assessment scores by dimension.

For each dimension of the reporting system, the systems assessment included a series of questions. Each question is scored on a scale that ranges from 1 to 3 (1 = no, not at all; 2 = yes, partly; 3 = yes, completely). Scores of 1 or 2 automatically generate a field for the assessor to note reasons for the score. The scores of individual questions are then averaged to produce a score for each dimension by each site included in the assessment, by reporting level, and across all reporting levels. The closer the scores are to 3, the stronger or more functional the reporting system is for that dimension. The systems assessment tool was programmed to automatically calculate the scores. Spider diagrams were used to visually display the overall results (an example is shown in **Figure 3**).

### Data verification

Data verification assessed reporting performance (the availability and completeness of reports) and the consistency of the data across the levels of the reporting system. In most cases, we were unable to assess timeliness because submission dates were not recorded. We programmed data tracing results to automatically populate, however this process required substantial customization to account for variations in data reporting systems before fieldwork and data cleaning to account for missing data after fieldwork. The reporting performance results presented the percentage of available and complete data collection and reporting forms by reporting level for the time-period assessed.

**Figure 4:**
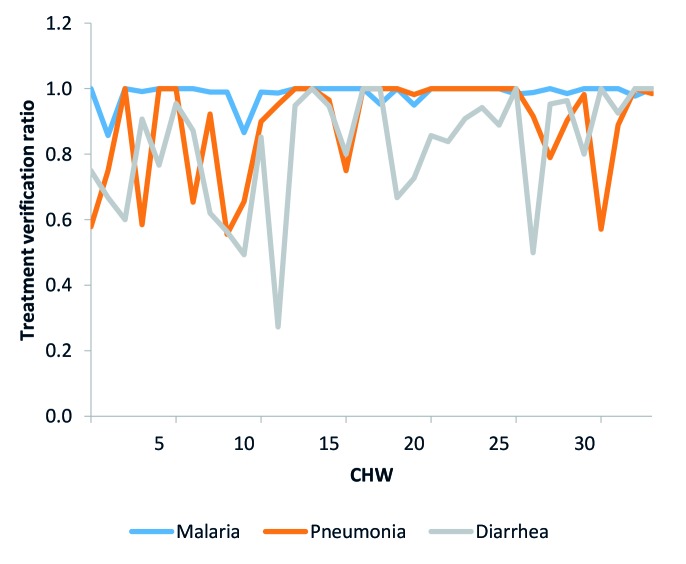
Treatment verification ratios example.

We calculated verification ratios for treatments recorded in the CHWs’ iCCM registers to assess if CHWs were appropriately filling out their registers when they recorded that they provided treatment to a sick child. The verification ratios compared the counts recorded in the treatment fields of the registers to the number of treatments appropriately recorded, by illness. We considered recorded treatments as appropriately recorded if the register entries included the appropriate corresponding symptomatic and diagnostic information. See **Figure 4** for an example of verification ratio trends for the three treatment indicators comparing recorded treatments to appropriately recorded treatments in CHW registers. This figure indicates that diarrhea in particular, and to a lesser extent pneumonia, were often not recorded appropriately.

**Figure 5:**
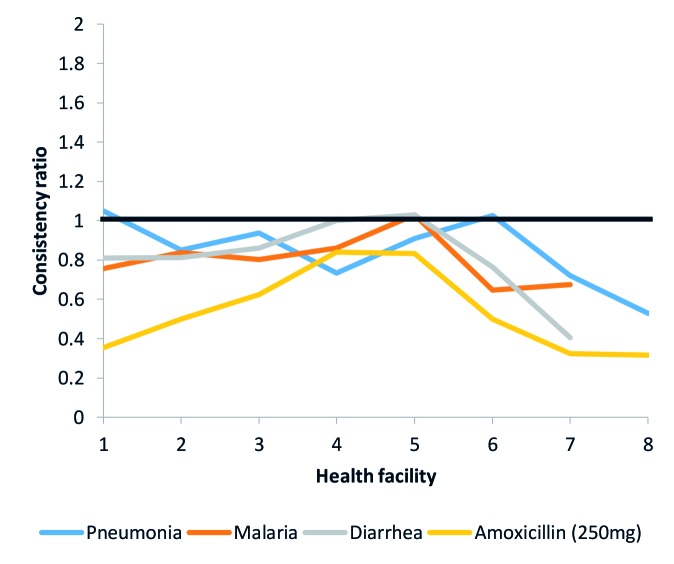
Facility reporting consistency ratios example.

We also calculated consistency ratios for each indicator to describe data consistency between reporting levels for the indicators traced in the DQA. These measures assessed data accuracy throughout the system. For these ratios, a value of 1 indicated agreement between the two counts being compared. Consistency ratios greater than 1 indicated under-reporting and consistency ratios less than 1 indicated over-reporting. Reasons the data may not match across forms include errors in calculation or transcription, different interpretations of data to be reported, missing or illegible data, or corrections made to errors that occurred on one form but were not corrected on the other form. For example, we compared the values reported in the health facility monthly reports by the health facilities included in the DQA with the sum of the values that CHWs reported in their summary reports for three treatment indicators and an amoxicillin stockout indicator (**Figure 5**). **Figure 5** shows that amoxicillin stockout was often over-reported to the next level.

In addition to the verification and consistency ratios, the DQA reports presented the counts verified in the CHWs’ iCCM registers and the counts reported by reporting tool to show the differences in counts between the various reporting tools and levels. These differences provide information about the magnitude, as well as the direction, of discrepancies and complements the consistency ratios, see **Table 4** for an example of the verification result counts for malaria treatment.

**Table 4:** Data verification results of counts and differences for malaria treatment

District	Facility	Selected facilities							
**Verified counts in CHW Registers**	**Counts in Summary Form of CHW Registers**	**Difference: Summary Form of CHW Registers minus CHW Registers**	**Counts in Facility Supervisor Summary Form**	**Difference: Facility Supervisor Summary Form minus Summary Form of CHW Registers**	**Counts in National HIS minus selected facilities**	**Difference: National HIS minus Facility Supervisor Summary Form**	**Difference: National HIS minus CHW Registers**
**1**	1	69	64	-5	64	0	64	0	-5
	2	275	299	24	327	28	327	0	52
**2**	3	1035	1066	31	991	-75	1092	101	57
	4	190	182	-8	183	1	245	62	55
**3**	5	105	107	2	106	-1	106	0	1
	6	121	125	4	120	-5	120	0	-1
**4**	7	620	836	216	829	-7	829	0	209
	8	982	1153	171	1150	-3	1170	20	188

CHW – community health worker, HIS – health interview survey

### Dissemination and recommendations

Dissemination of findings and provision of recommendations were important aspects of the DQAs. At the conclusion of each DQA, we conducted a debriefing with initial results in-country for key stakeholders. A comprehensive DQA report, which included recommendations based on the findings, was drafted and reviewed by WHO and grantees prior to the finalization. WHO then facilitated dissemination in-country to present and discuss recommendations with the MOH. ICF supported some grantees to develop specific action plans to address DQA recommendations. **Table 5** presents some examples of recommendations that resulted from the DQAs. See Yourkavitch et al. (2018) for discussion on the steps taken by MOHs and grantees to implement recommendations [26].

**Table 5:** Examples of recommendations from DQAs

Recommendations
*Develop a systematic training plan for staff at all levels.* A training plan should be developed to ensure that all staff at different levels of the iCCM reporting system receive the necessary training they need to be effective in their work. The training plan will ensure ongoing capacity building of CHWs, CHW supervisors, and M&E officers to improve the accuracy of the data they collect and report. The training plan should also include scheduled refresher trainings on data quality sessions during monthly or quarterly meetings. A good start would be a review of the existing training plans mentioned during the DQA interviews, then adapt and customize them for the national, district, facility and community levels.
*Support the MOH iCCM unit with the development of written guidance and procedures.* The systems assessment found data management practices to be the weakest point of the M&E system, primarily because written procedures do not exist to address issues with late, incomplete, inaccurate, and missing reports. Despite this, all of the licensed nurses interviewed could correctly describe what should be done in case of problems with the data. ICF recommends that the grantee support the MOH iCCM unit in the development, documentation, and implementation of standard procedures for each level of the CHW reporting system.
*Create a standardized process to ensure that information is properly transferred on duplicate and triplicate copies of registers and summary forms*. Often, the information recorded on the duplicate or triplicate copies is not an accurate reflection of what was recorded on the original copy. Reasons for such mistakes include the use of pencil, misalignment or improper placement of carbon copy paper, or failure to use a cardboard divider when recording information. This standard process should also be emphasized during supervisory visits.
*Track CHW-level consumption information separately.* Currently, CHW data related to medical supplies and commodities consumption are aggregated at the health facility level. These consolidated data do not flow to the district or provincial level. Hence, there is no mechanism for making data-driven estimates for CHW consumption and supply. The MOH should develop a system that enables consumption data specific to the CHW level to be reported through the CHW program and general supply chain.

### iCCM data quality assessment toolkit and guidance

Throughout the DQA process, RAcE grantees and MOH staff increasingly recognized the importance of data quality and the need to routinely and rapidly measure the quality of iCCM data. At the request of WHO, ICF developed an iCCM DQA Toolkit to address this need. We designed the iCCM DQA Toolkit for MOH or implementing partner staff at the national and sub-national levels to conduct periodic data quality checks. Specifically, the toolkit is designed to assess the data recorded by CHWs as they are aggregated through the reporting system, from the community to the national level. The toolkit includes two Excel-based tools, the systems assessment tool and the data-tracing tool.

- The systems assessment tool includes a set of modules, one for each level of the data reporting system. The DQA team records and scores each item in the module, and the tool generates a scorecard to display the results.
- The data-tracing tool includes data collection and analysis worksheets for tracing selected indicators through the iCCM data reporting system. The DQA team uses the tool to review and collect information from the iCCM data collection and reporting tools at each level of the data reporting system at the sites selected for assessment. The tool then uses these data to calculate measures of data availability, completeness, and consistency as the data are aggregated through the iCCM data reporting system. A series of charts and tables included in the tool display these measures.

Development of the iCCM DQA Toolkit was a participatory process. After ICF developed the draft toolkit, we piloted both the systems assessment and data tracing tools in Abia State, Nigeria. We conducted the pilot over a period of four days. Site visits took place in three health facilities, an LGA office (intermediate aggregation level), and the State Ministry of Health. The first day included holding a toolkit orientation with MOH staff and conducting the systems assessment with the LGA focal point and the State and Federal officers. Facility visits took place on the second and third days of the pilot. At the facilities, we conducted the systems assessment with the CHW supervisor and used the data tracing tools to extract data from CHW, CHW supervisor, and LGA data collection and reporting tools. The fourth day of the pilot included entering the field data into the Excel tool, reviewing the data analysis process, and gathering feedback on the overall process and tools with MOH and LGA staff.

The toolkit has an accompanying guidance document, which provides detailed information on various aspects of the toolkit and guidance on DQA implementation. Specifically, the guidance document describes the purpose and structure of the iCCM DQA toolkit; considerations for determining personnel and logistics, selecting the sample, and preparing for fieldwork; instructions for adapting the tools; guidance on how to use the tools to implement a DQA; and guidance for analyzing, visualizing, and interpreting the data collected during the DQA. The complete toolkit and accompanying guidance document are available on http://ccmcentral.com/. It is important to note that some of the elements described above as part of the RAcE DQAs are not included in the iCCM DQA Toolkit or guidance; specifically there are not qualitative data collection tools nor is there provision for the calculation of verification ratios from iCCM registers. We excluded these two elements to facilitate a rapid and routine process.

## DISCUSSION

Through RAcE, ICF conducted several comprehensive DQAs of routine iCCM data generated by CHWs. The DQAs evaluated the data reporting systems in place and provided recommendations on how to improve data quality. Details on some of the DQAs conducted under RAcE have been published elsewhere [21,23]. In general, however, there is limited literature on the process of conducting DQAs, specifically for data generated at the community level. Such accounts are especially important for iCCM programs that have additional challenges as they rely on community health systems that are overstretched and CHWs that often times are volunteers with limited formal education or training and low literacy levels. The communities that iCCM programs primarily target face other resources challenges such as poor infrastructure, transport, roads, electricity and mobile network/internet coverage challenges that place additional strains on the reporting system. However, the data collected by CHWs are critical to inform decisions about implementation and to demonstrate program successes and challenges.

ICF developed DQA tools to assess the iCCM data reporting system and to trace iCCM indicators through this system. Because data reporting tools and flows vary by country, a critical first step in conducting a DQA is to understand the data flow and reporting tools and customize DQA tools accordingly. The iCCM DQA Toolkit provides a starting point for designing a DQA for a specific iCCM reporting system. The DQA tools have proven to be effective, efficient, and generated recommendations to facilitate improving data quality. The DQAs conducted through RAcE took 2-3 weeks to complete, which could be burdensome for MOH staff. The iCCM DQA Toolkit is streamlined to be less burdensome and enable countries to more readily identify points in the reporting system where data quality is compromised and which dimensions of the reporting system require strengthening. We conducted the pilot exercise in Abia State, Nigeria in 3.5 days.

The toolkit includes systems assessment questions that elicit actionable responses, and the data tracing tool is simplified to focus on one indicator for each of the three iCCM-focus illnesses. The systems assessment examines the functional dimensions of the data reporting system that are needed to ensure data quality. Adapted from the MEASURE Evaluation conceptual framework, the RAcE DQA tools examined five dimensions: M&E structures, functions and capabilities; indicator definitions and reporting guidelines; data collection and reporting forms and tools; data management processes; and links with national reporting systems. The conceptual framework has since been updated to include a sixth component, use of data for decision making [2]. Elements to assess if data are being used for decision making include visualizing data through the development of charts, graphs, maps or other resources; ability to interpret and analyze data; access to guidance or technical assistance on data use; presentation and dissemination of data to key stakeholders; and evidence of decisions taken based on the analyzed data and results [2].

The toolkit also provides guidance on modifications national and sub-national MOH staff can make to tailor the tools to meet their iCCM program needs and yield DQA findings that improve data quality, strengthen reporting systems, and inform decision-making. Although there are several toolkits and guidance documents in existence [2,27-29], these tools to do not factor in the community level and thus, the feedback loop to the lowest (community) level and a thorough assessment of the data collection and reporting systems at the community level is often missing.

## CONCLUSIONS

The process of implementing a DQA not only improves data quality, but is also beneficial to the DQA team. We recommend that staff at the national or sub-national level use the iCCM DQA Toolkit for periodic data quality checks or as part of routine supervision of iCCM programs. In this vein, the DQA would serve multiple purposes including capacity building, supervision, and identification of challenges or bottlenecks by those in a position to implement changes.
